# Intra-articular delivery of adipose derived stromal cells attenuates osteoarthritis progression in an experimental rabbit model

**DOI:** 10.1186/ar4156

**Published:** 2013-01-29

**Authors:** Giovanna Desando, Carola Cavallo, Federica Sartoni, Lucia Martini, Annapaola Parrilli, Francesca Veronesi, Milena Fini, Roberto Giardino, Andrea Facchini, Brunella Grigolo

**Affiliations:** 1 Laboratory of Immunorheumatology and Tissue Regeneration, Rizzoli Orthopaedic Institute, Via di Barbiano 1/10, Bologna, 40136, Italy; 2 Laboratory RAMSES, Rizzoli Orthopaedic Institute, Via di Barbiano 1/10, Bologna, 40136, Italy; 3 Laboratory of Preclinical and Surgical Studies, Rizzoli Orthopaedic Institute, Via di Barbiano 1/10, Bologna, 40136, Italy; 4 BITTA Laboratory, Rizzoli Orthopaedic Institute, Via di Barbiano 1/10, Bologna, 40136, Italy; 5 Department of Clinical Medicine, University of Bologna, Via Massarenti 9, Bologna, 40136, Italy

## Abstract

**Introduction:**

Cell therapy is a rapidly growing area of research for the treatment of osteoarthritis (OA). This work is aimed to investigate the efficacy of intra-articular adipose-derived stromal cell (ASC) injection in the healing process on cartilage, synovial membrane and menisci in an experimental rabbit model.

**Methods:**

The induction of OA was performed surgically through bilateral anterior cruciate ligament transection (ACLT) to achieve eight weeks from ACLT a mild grade of OA. A total of 2 × 10^6 ^and 6 × 10^6 ^autologous ASCs isolated from inguinal fat, expanded *in vitro *and suspended in 4% rabbit serum albumin (RSA) were delivered in the hind limbs; 4% RSA was used as the control. Local bio-distribution of the cells was verified by injecting chloro-methyl-benzamido-1,1'-dioctadecyl-3,3,3'3'-tetra-methyl-indo-carbocyanine per-chlorate (CM-Dil) labeled ASCs in the hind limbs. Cartilage and synovial histological sections were scored by Laverty's scoring system to assess the severity of the pathology. Protein expression of some extracellular matrix molecules (collagen I and II), catabolic (metalloproteinase-1 and -3) and inflammatory (tumor necrosis factor- α) markers were detected by immunohistochemistry. Assessments were carried out at 16 and 24 weeks.

**Results:**

Labeled-ASCs were detected unexpectedly in the synovial membrane and medial meniscus but not in cartilage tissue at 3 and 20 days from ASC-treatment. Intra-articular ASC administration decreases OA progression and exerts a healing contribution in the treated animals in comparison to OA and 4% RSA groups.

**Conclusions:**

Our data reveal a healing capacity of ASCs in promoting cartilage and menisci repair and attenuating inflammatory events in synovial membrane inhibiting OA progression. On the basis of the local bio-distribution findings, the benefits obtained by ASC treatment could be due to a trophic mechanism of action by the release of growth factors and cytokines.

## Introduction

Osteoarthritis (OA) is one of the most common and widespread rheumatic disease among adults with a significant and negative impact on patient quality of life [1]. OA affects the whole joint and is characterized by inflammation, bone remodeling and progressive destruction of the articular cartilage components with following functional disability [2]. In OA, the earliest changes in cartilaginous tissue appear at the joint surface where mechanical forces are greatest. Chondrocytes in OA cartilage, especially those arranged in clonal clusters, exhibit cytokine and chemokine receptors, increase production of matrix proteins and matrix degrading enzymes leading to a modulation of inflammatory and catabolic responses [3]. The altered homeostasis of the extracellular matrix (ECM) macromolecules in cartilage tissue during OA leads to an increased enzymatic activity of metalloproteinases (MMPs) [4] and enhances the synthesis of pro-inflammatory molecules [5,6] with following pain and joint instability. The currently available treatments for OA are effective only on a short term and there is a largely unmet medical need for durable disease-modifying treatments [7]. Established therapies for OA include mainly preventive measures such as weight control, exercise or pharmacologic approaches which usually consist of analgesic therapy, including acetaminophen, salicylates and non-steroidal anti-inflammatory drugs [8-10]. The feasibility of using mesenchymal stem cells (MSC) from bone marrow or other tissue sites, based on their capacity to influence and regulate different stages of cartilage repair, is a challenge of considerable appeal to clinicians. In particular, studies using animal models have shown promising results following MSC therapy for the treatment of musculoskeletal injuries [11]. In recent years, several studies indicate that the therapeutic properties of MSC are due not only to their capacity to differentiate but also to their ability to release growth factors that regulate the immune response in a paracrine manner [12,13]. Other multipotent cell types have been found in different compartments, including adipose tissue, also known as adipose derived stromal cells (ASCs) [14]. ASCs can easily be obtained from liposuction waste in large quantities with little donor site morbidity and are known to differentiate along selected lineage pathways in response to specific growth factors and environmental cues [15-18]. ASCs represent valid candidates to promote cartilage and menisci healing due to their ability to release biologically chondrogenic active factors, such as transforming growth factor-β1 (TGF-β1) and bone morphogenetic protein 4 (BMP-4) [19], anti-fibrotic and anti-apoptotic growth factors [20,21]. Moreover, some studies have provided insights into the role of ASCs in suppressing immunoreactions as MSC [22,23], suggesting their possible use in decreasing local inflammation in several musculoskeletal diseases. Different musculoskeletal treatments with ASCs have already been reported with encouraging results for the regeneration of cartilage and bone, even if the mechanism of action is not clearly defined yet [24]. Up to date, functional data on the role of ASCs in the care of osteoarthritis are, however, still scarce [25-28]. The main aim of the current study was to explore the efficacy of an intra-articular injection of ASCs in preventing cartilaginous and menisci damages and attenuating inflammation in synovial membrane following the onset of OA in a rabbit model. In general, our findings revealed a positive effect of ASCs in promoting cartilage and menisci healing and contrasting inflammatory processes in the synovial membrane.

## Materials and methods

### Rabbits

Adult male New Zealand rabbits (age: 12 months old, body weight: 4 ± 0.5 Kg) were used. European and Italian laws on animal experimentation were strictly followed throughout the study. OA was induced surgically by bilateral anterior cruciate ligament transection (ACLT) [29,30]. Adipose tissue was harvested from the inguinal zone for ASCs isolation. A total of 2 × 10^6 ^and 6 × 10^6 ^ASCs were re-suspended in 4% rabbit serum albumin (RSA) (Sigma Aldrich, St. Louis, MO, USA) and administered by an intra-articular injection into the hind limbs after OA onset, eight weeks from ACLT. Four percent RSA was used as a control group. A small number of animals were designed to monitor the fate of the cells at 3 and 20 days after ASC injection. Animals were sacrificed respectively at short- (16 weeks) and long- (24 weeks) term follow-ups from ASC administration to investigate their effect on different compartments of the knee joint. To this end, femoral condyles, meniscal and synovial tissues were harvested. Table 1 shows the groups involved in the experimental study.

**Table 1:** Description of the experimental design

Groups	Number of animals	Treatments	Follow-up
**Sham group**	3	Sham operation	8 wks
**OA group**	9	ACLT	8, 16, 24 wks
**4% RSA group**	24	4% RSA	16 to 24 wks
**2 × 10^6 ^ASC group**	24	2 × 10^6 ^ASCs	16 to 24 wks
**6 × 10^6 ^ASC group**	24	6 × 10^6 ^ASCs	16 to 24 wks
**Biodistribution group**	9	labeled - ASCs	3, 7, 20 days

ACLT, anterior cruciate ligament transection; ASCs, adipose stem cells; OA, osteoarthritis; RSA, rabbit serum albumin; wks, weeks

### Osteoarthritis model

For the ACLT procedure, a 2 cm skin and capsular incision was carried out and right and left ACLs were exposed through a medial para-patellar cut. To achieve optimal visualization of the ACL, the patellar bone was displayed laterally and the knee was placed in full flexion. To avoid spontaneous reattachment, the ACLT was associated to the removal of a small fragment of tissue between the two ligament stumps. The incision was sutured in a routine fashion. After each operation, an antibiotic (Flumequine, Sigma) and analgesic (Ketoprofene, Rhone-Poulenc-Rorer, Sanofi Aventis, Strasbourg, France) therapy was administered immediately after surgery and for two days thereafter. All surgical procedures were performed under general anesthesia and sterile conditions.

### ASC isolation and growth OK

Adipose tissue was harvested from the rabbit inguinal zone and treated with 0.4 U/ml NB4 collagenase standard grade (Serva Electrophoresis, GmbH, Heidelberg, Germany) to isolate ASCs. The stromal vascular fraction (SVF) containing ASCs was re-suspended in α-MEM (Gibco, Carlsbad, CA, USA) supplemented with 1 U/ml heparin (Sigma, St Louis, MO, USA), 2% platelet growth factor enriched plasma (PGFEP) [31] and 0.05 g/ml penicillin G (Gibco). Initially, ASCs were plated at a density of 4,000 cells/cm^2 ^and cultured for a few days. Cells were then harvested and seeded at a density of 2,000 cells/cm^2 ^for the expansion. Viability was evaluated at SVF isolation and expansion by the Trypan Blue dye exclusion method. The selection of ASCs was carried out on the basis of their ability to adhere to the plastic, to form colonies and to differentiate into chondrogenic and osteogenic lineages [15,32]. The number of population doublings (PD) was calculated to verify rabbit ASCs growth during the culture period.

### ASCS administration

A total of 2 × 10^6 ^(cell density: 6 × 10^6 ^cells/ml) and 6 × 10^6 ^(cell density: 18 × 10^6^/ml) autologous ASCs at passage 1 in 4% RSA were prepared upon sterile conditions in 1 ml syringes and delivered by an intra-articular injection into the hind limbs at OA onset (eight weeks). The needle was inserted into the knee joint posterior to the lateral edge of the patella at the junction of the femur and tibia to avoid damage to the articular cartilage. The sample was injected into the joint capsule and the knee was flexed. The rabbit was held in this position for a few minutes before recovery. Post-operative course and long-term adverse events were monitored.

### Local bio-distribution of ASCS

The fate of the ASCs was monitored by the local bio-distribution evaluation at 3 and 20 days from ASC administration. ASCs were labeled *in vitro *by 6 μM chloro-methyl-benzamido-1,1'-dioctadecyl-3,3,3'3'-tetra-methyl-indo-carbocyanine per-chlorate (CM-Dil) dye (Molecular Probes, Carlsbad, CA, USA) [33] as indicated by the manufacturer. A total of 6 × 10^6 ^labeled-ASCs were injected in the hind limbs and un-labeled cells in the contra-lateral ones, as the control. Cells were monitored *in vitro *in parallel with the *in vivo *experiments (3 and 20 days) to evaluate ASCs' viability, their doubling time and their differentiation potential. Animals were sacrificed and the different tissues (femoral condyle, tibial plateau, synovial membrane, menisci, ligament and articular capsule) were processed for histology by paraffin embedding. Sections were analyzed by epi-fluorescent microscopy Eclipse 90i (Nikon, Melville, NY, USA) by using 4',6-diamidino-2-phenylindole (DAPI) and TRITC filters to evaluate nuclear component and labeled-ASCs, respectively.

### Macroscopic imaging and histopathology

Macroscopic assessment of the knee joints from the animals that underwent ACLT was performed by India ink staining (Higgings Waterproof Drawing Ink, Eberhard Faber, Lewisburg, TN, USA) to assess cartilage lesions. Macroscopic assessments were also performed in the groups treated with 4% RSA and ASCs at 16 and 24 weeks [34]. The histo-morphometric evaluations were performed by image analysis Qwin v 2.4.4 software (Leica, Imaging Systems, Cambridge, UK) on osteo-chondral specimens embedded in metacrylate. Quantitative measurements of cartilage thickness (CT) and fibrillation index (FI) in ASC-treated groups and 4% RSA were carried out at both experimental times. All the analyses were performed by two blinded investigators according to the indications provided by Papaioannou and Pastoureau [35,36]. To perform histological analysis, synovial membrane, menisci and femoral condyles were placed in 10% neutral buffered formalin and osteo-chondral specimens were decalcified for three weeks at room temperature (RT). Specimens were paraffin embedded and thin sections (5 μm) were taken. Hematoxylin/Eosin and Safranin-O/Fast Green (Sigma) stainings were used to assess general morphology and proteoglycans/collagen content in synovial, meniscal and cartilaginous tissues respectively. Semi-quantitative analyses using appropriate scoring systems were used to evaluate the cartilaginous and synovial tissues [37]. In particular, the assessment of cartilage tissue was carried out with Laverty's scoring system which takes into account four parameters: Safranin-O/Fast Green staining, cartilage structure, chondrocyte density and cluster formation. It has a range from 0 to 24 where 0 translates to healthy cartilage while 24 to severe cartilage lesions. The assessment of synovial tissue was performed with a semi-quantitative scoring system that comprises the histological features of synoviopathy in OA, including the synoviocytes (proliferation, hypertrophy), the inflammatory state and the synovial stroma (hyperplasia, proliferation of blood vessels, proliferation of fibroblasts, cartilage/bone detritus). It has a range from 0 to 30 where 0 translates to a normal white, semi-translucent smooth tissue and 30 indicates severe proliferation, hypertrophy, inflammation and hyper-vascularity. All the evaluations were performed by two blinded researchers with an Eclipse 90i microscope (Nikon).

### Immunohistochemistry

The analyses were carried out to evaluate type I and II collagens, MMP-1, MMP-3 and TNF-α on cartilage, synovial and meniscal specimens. Appropriate un-masking procedures using specific treatments, including hyaluronidase and pronase for collagens and citrate buffer solution for MMPs and TNF-α, followed by blocking steps were carried out. Fixed samples were incubated at RT with mouse monoclonal antibodies directed against type I collagen (2 μg/ml) (Sigma), type II collagen (2 μg/ml) (Hybridoma Bank, Department of Biological Sciences, University of Iowa, Iowa City, IA, USA), MMP-1 (5 μg/ml) (Chemicon, Temecula, CA, USA), MMP-3 (5 μg/ml) (Chemicon) and TNF-α (2 μg/ml) (Santa Cruz Biotechnology, Inc., Santa Cruz, CA, USA) respectively. Biotinylated secondary antibody and alkaline-labelled streptavidin (Biocare Medical, Walnut Creek, CA, USA) were used. The reactions were developed using Fast Red substrate (Biocare Medical). Negative controls were performed either by omitting the primary antibodies or using an isotype-matched control. Six microscopic fields (100 × magnification) relative to the anterior, central and posterior regions in cartilage tissue were used to perform a semi-quantitative analysis of immunohistochemistry. A semi-quantitative method that assigns immunohistochemistry values as a percentage of positive cells (collagen I, MMPs, TNF-α) or positive area (extracellular matrix) (collagen II) was provided for a complete assessment of protein expression, with maximum scoring being 100%. The analysis was performed by two blinded investigators using Red/Green/Blue (RGB) with Software NIS-Elements and Eclipse 90i microscope (Nikon).

### Statistical analysis

Statistical analysis was carried out using the Statistical Package for the Social Sciences (SPSS Inc., Chicago, IL, USA) software version 15.0 (SPSS Inc.). Data for Laverty scores, CT, FI, number of cluster formation and immunohistochemical quantifications were expressed in terms of 95% confidence intervals (CI) of the mean and/or mean ± standard deviation (SD). The general linear model (GLM) with Sidak correction for multiple comparisons was used to assess the influence of the kind of treatment and follow-up on the different parameters listed above. The Wilcoxon signed rank test was used instead for paired comparisons in order to evaluate the effects exerted by each group at the different experimental times.

## Results

### Development of mild grade of osteoarthritis

**Figure 1:**
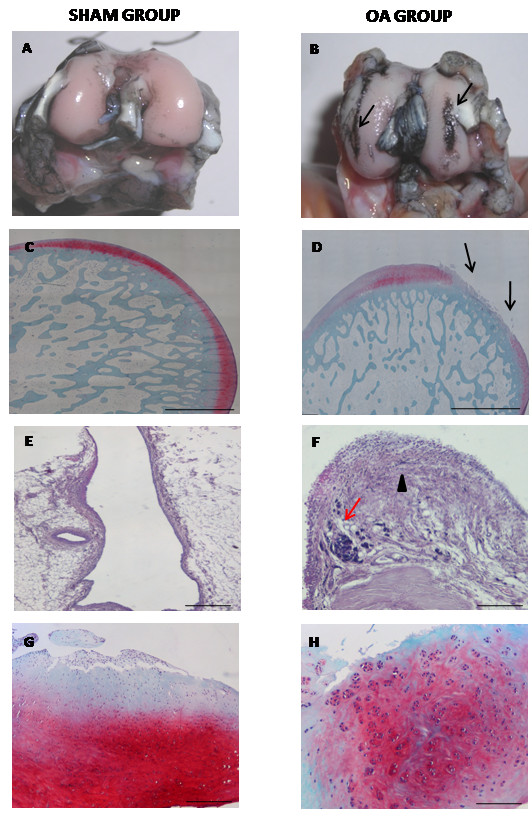
**Degenerative changes in different articular compartments in sham and OA groups at eight weeks**. (**A, B**) India ink staining of medial femoral condyle (MFC) of representative specimens. Arrows, fibrillation processes (**C, D**) Safranin-O/Fast Green staining of cartilage tissue of representative specimens. Arrows: Fibrillation and delamination processes. (**E, F**) Hematoxylin/Eosin staining of synovial membrane of representative specimens. Black arrows, Thickening of lining layer; Red arrows, Inflammatory cells. (**G, H**) Safranin-O/Fast Green staining of medial meniscus of representative specimens. Scale bars in C, D = 1 mm; E-H = 100 μm.

All the animals developed a mild grade of OA at eight weeks after ACLT without any re-attachment of the ligament. Macroscopic analysis gave evidence of varying degrees of OA features in the OA group, with evident black patches due to the uptake of India ink for cartilage softening and fibrillation processes (Figure 1B) [38]. Histological findings revealed a series of OA changes, including cartilage fibrillation and erosion, peri-articular osteophytosis and moderate reduction in meta-chromatic staining in the cartilage tissue (Figure 1D) [30,38]. As concerns synovial tissue, thickening of the lining layer and presence of inflammatory cells were observed by histological analysis at eight weeks (Figure 1F). The investigations on menisci revealed fibrillation processes in the area of femoral attachment and an increased presence of cell clusters by means of histological analysis (Figure 1H).

### Local biodistribution of CM-Dil labeled ASCS into the OA knee joint

**Figure 2:**
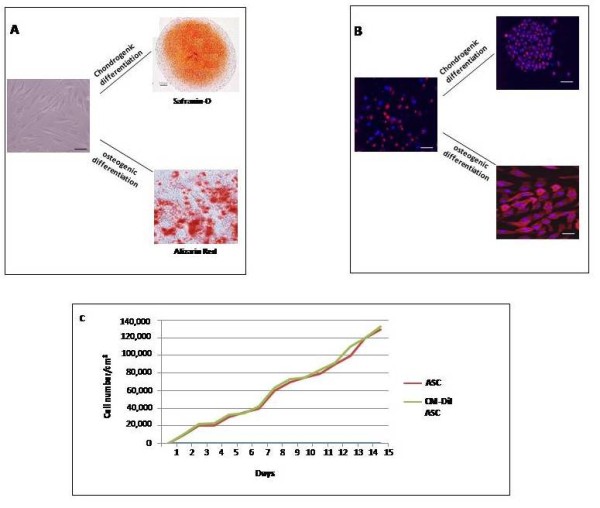
**Chondrogenic and osteogenic differentiation of rabbit adipose stem cells (ASCs) *in vitro***. (**A**) Chondrogenic and osteogenic differentiation of rabbit ASCs labeled with CM-Dil *in vitro*. (**B**) Growth curves of unlabeled and labeled ASCs (**C**).

**Figure 3:**
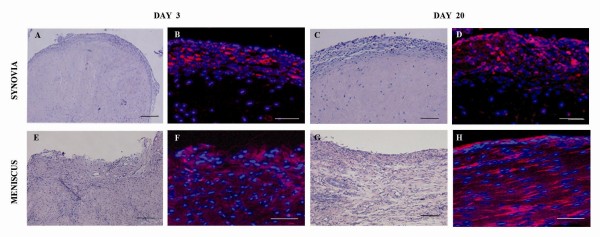
**Biodistribution of CM-Dil labeled ASCs**. Detection of labeled-ASCs in synovial lining layer (**A-D**) and medial meniscus (**E-H**) of representative specimens at 3 and 20 days from ASC administration under bright field (A, C, E, G) and epi-fluorescence (B, D, F, H). Blue staining = nuclei; Red staining = CM-Dil labeled-ASCs. Scale bars = 500 μm.

The CM-Dil dye provided *in vitro *a uniform labeling of the cells (90%) as assessed by fluorescence microscopy. The staining did not affect the ASCs' viability (95%), cell doubling (2.09 PD at passage 1 and 1.7 PD at passage 3) and differentiation potential into chondrogenic and osteogenic lineages (Figure 2). Regardless of the bio-distribution *in vivo*, ASCs were clearly detected in the synovial membrane and medial meniscus at 3 and 20 days (Figure 3). In particular, the presence of ASCs in the lining layer of synovial membrane and a diffuse distribution along the thickness of fibro-cartilaginous and vascular areas of the medial meniscus were noticed. No cell engraftment was observed in the anterior cruciate ligament, in the articular cartilage from the medial or lateral femoral condyle and in the tibial plateau at any of the experimental times evaluated (data not shown).

### ASCS treatment prevents cartilage destruction

**Figure 4:**
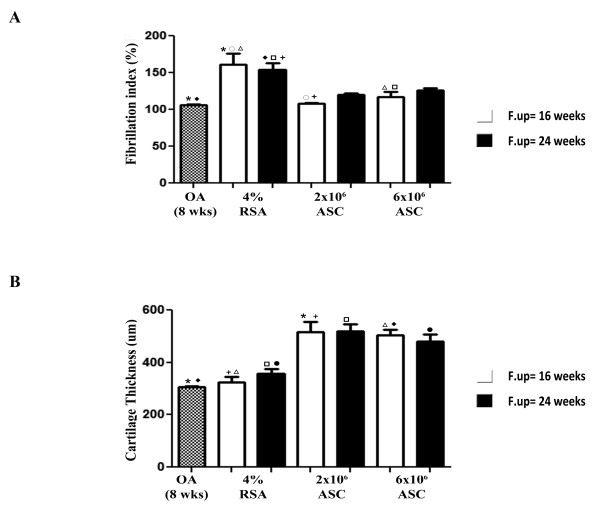
**Quantitative assessment of disease progression in the different groups**. (**A**) Fibrillation index. (**B**) Cartilage thickness. Data are reported in terms of mean ± SD. Statistical values of at least *P *< 0.01 were observed in all the comparisons.

**Figure 5:**
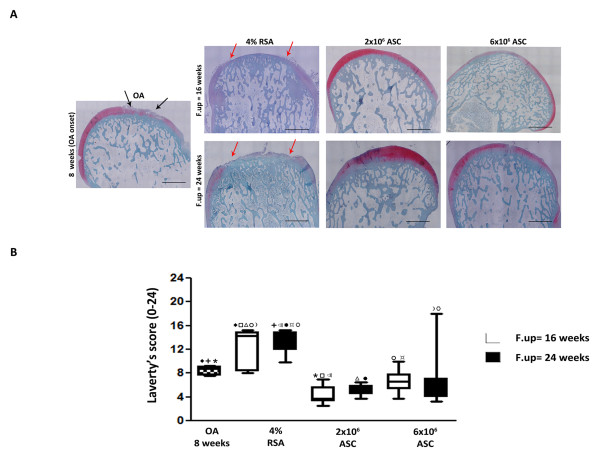
**Histological evaluation of medial femoral condyle in OA, 4% RSA and ASC-treated groups**. (**A**) Safranin-O/Fast Green staining of representative specimens. Black arrows, fibrillation and delamination processes. Red arrows, Erosion processes. Scale bars = 1,000 μm. (**B**) Laverty's score. Data are reported in terms of 95% confidence intervals. Statistical values of at least *P *< 0.01 were observed in all the comparisons.

A glossy, white cartilage with no degenerative noticeable macroscopic evidence was observed in the ASCS-treated groups at both experimental times. Cartilage softening and fibrillation were instead observed in the OA and 4% RSA groups particularly in the medial regions (data not shown). Histo-morphometric analyses provided further information on the status of the structure of cartilage tissue. Among the pathological changes recorded during OA, an increase of FI was observed in OA group compared to 4% RSA at both 16 and 24 weeks (*P *< 0.01). A significant reduction of FI was noticed in both ASCs treated groups at 16 weeks compared to 4% RSA at both experimental times (*P *< 0,01), indicating a protective role of ASCs in reducing fibrillation processes (Figure 4A). The intra-articular delivery of 2 × 10^6 ^ASCs revealed an increase of CT values respect the OA group and 4% RSA at 16 and 24 weeks (*P *< 0.01) (Figure 4B). Based on visual assessment of the histological staining, there were significant, overall effects of ASCs in the healing of cartilaginous tissue which displayed some hyaline features with a smooth surface, a regular cellular arrangement and a high proteoglycan content leading to a functional cartilage tissue (Figure 5A, B). In particular, 2 × 10^6 ^ASC treatment revealed a significant decrease of Laverty's score at 16 weeks compared to the OA group (*P *< 0.01), which instead gave evidence of a series of changes, including chondrocyte and proteoglycan loss, fibrillation and delamination processes. A progression of the pathology, was also noticed in the 4% RSA group at 16 and 24 weeks compared to OA (*P *< 0.01). In general, ASC administration determined a decrease of Laverty's scores compared to 4% RSA (*P *< 0.01) at both experimental times.

### ASCS treatment inhibits the thickening of lining layer in synovium and reduces cluster formation in menisci

**Figure 6:**
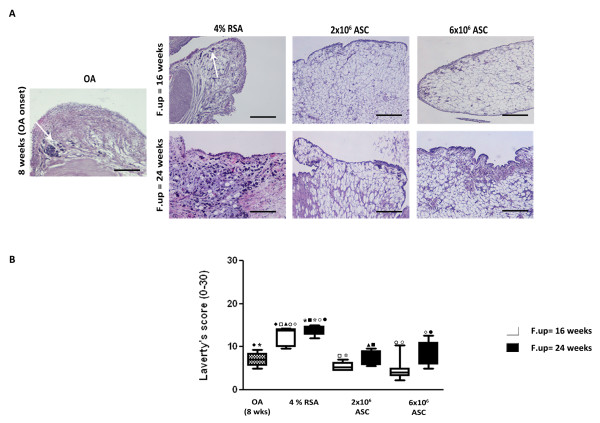
**Histological evaluation of synovial membrane in OA, 4% RSA and ASC-treated groups**. (**A**) H/E staining of representative specimens. Scale bars = 100 μm. (**B**) Laverty's score. Data are reported in terms of the 95% confidence intervals. Statistical values of at least *P *< 0.01 were observed in all the comparisons.

**Figure 7:**
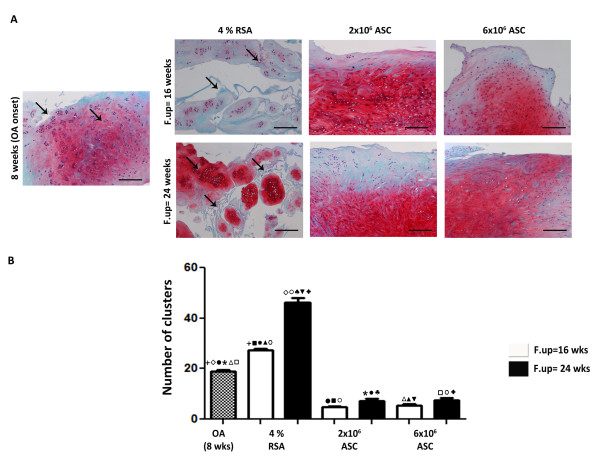
**Histological evaluation of menisci in OA, 4% RSA and ASC-treated groups**. (**A**) Safranin-O/Fast Green staining of representative specimens. Scale bars = 100 μm. (**B**) Numbers of cluster formation. All the data are reported as 95% confidence intervals. Statistical values of at least *P *< 0.01 were observed in all the comparisons.

Synovial tissue after ACLT in OA group displayed at eight weeks thickening of the lining layer and evidence of infiltration by inflammatory cells. A progression of the pathology was detected in the 4% RSA group at 16 and 24 weeks with an increase of Laverty's score compared to the OA group (*P *< 0.01). ASC administration (2 × 10^6 ^and 6 × 10^6^) significantly decreased Laverty's score at 16 and 24 weeks compared to the 4% RSA and OA groups (*P *< 0.01). The protective effects exerted by the ASCs were noticed overall in reducing the thickness of the lining layer and the infiltration of inflammatory cells in the sub-synovium. In particular, best results were noticed in the 6 × 10^6 ^ASC-treated group at 16 weeks compared to 24 weeks (*P *< 0.01) (Figure 6A, B). An histological analysis on menisci was performed in order to determine if ASC treatment had an effect on the meniscal compartment. A significant increase of cell cluster was noticed in the 4% RSA at 16 and 24 weeks compared to the OA group (*P *< 0.01). The 2 × 10^6 ^and 6 × 10^6 ^ASC-treated groups displayed a well organized tissue with a low number of cell clusters at 16 and 24 weeks compared to the OA and 4% RSA groups at both experimental times (*P *< 0.01) (Figure 7A, B).

### ASCS treatment reduces matrix degrading enzymes and TNF-α in cartilage matrix

**Figure 8:**
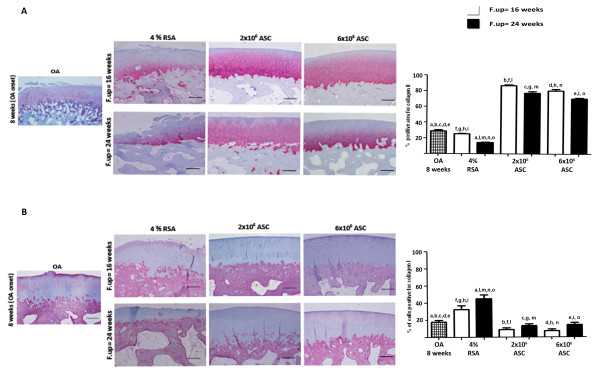
**Immunohistochemical analysis for collagen II and I in cartilage**. Photomicrographs of representative specimens evaluated for type II (**A**) and I (**B**) collagens in medial femoral condyle of OA, 4% RSA and ASC-treated groups. Data are reported as mean ± SD. Scale bars = 100 μm. Statistical values of at least *P *< 0.01 were observed in all the comparisons.

**Figure 9:**
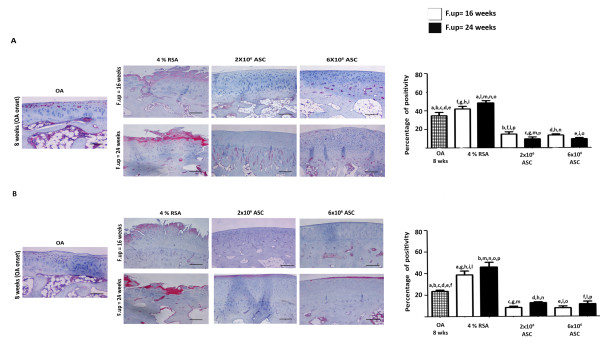
**Immunohistochemical analysis for MMP-1 and TNF-α in cartilage**. Photomicrographs of representative specimens evaluated for MMP-1 (**A**) and TNF-α (**B**) in medial femoral condyle of OA, 4% RSA and ASC-treated groups. Data are reported as mean ± SD. Scale bars = 100 μm. Statistical values of at least *P *< 0.01 were observed in all the comparisons.

Since the degradation of cartilage matrix represents a key event in the development of OA, we decided to test the effect of ASC treatment on catabolic and inflammatory molecules involved in the OA onset. We first investigated the typical hyaline marker, collagen type II, detecting a decrease of this molecule in the 4% RSA at 24 weeks in respect to the OA group (*P *< 0.01). ASC treatment gave evidence of a chondro-protective effect, promoting the expression of a great amount of type II collagen in the cartilage tissue in respect to the OA and 4% RSA groups (*P *< 0.01). In particular, high percentages of positive areas in the 2 × 10^6 ^and 6 × 10^6 ^ASC-treated groups at 16 and 24 weeks were detected. A reduced expression of collagen type II was noticed in 4% RSA, 2 × 10^6 ^and 6 × 10^6 ^ASCs at 24 weeks compared to 16 weeks (*P *< 0.01) (Figure 8A). Collagen type I, a fibro-cartilaginous marker, reported an intense positivity at cellular level in the OA group, particularly at the superficial level in cartilage matrix. The 4% RSA group displayed an increased expression of type I collagen particularly at 24 weeks compared to the OA group (*P *< 0.01). A reduction of collagen type I was detected in the ASC-treated groups at short- and long-term follow-ups with respect to the 4% RSA group (*P *< 0.01) (Figure 8B). A moderate expression of MMP-1 was noticed in the OA group, particularly in the superficial layer of cartilage. An increased expression of this protein was observed in the 4% RSA group. By contrast, ASC-treated groups showed a low expression for MMP-1 compared to the OA group and 4% RSA group at 16 and 24 weeks (*P *< 0.01) (Figure 9A). The OA group displayed a moderate expression of TNF-α which increased progressively in 4% RSA at 16 and 24 weeks (*P *< 0.01). A reduction of TNF- α expression was detected in both the ASC-treated groups at 16 weeks and 24 weeks with respect to the OA and 4% RSA groups (*P *< 0.01) (Figure 9B).

### ASCS treatment inhibits MMP-1 and TNF-α expression in synovial membrane and menisci

**Figure 10:**
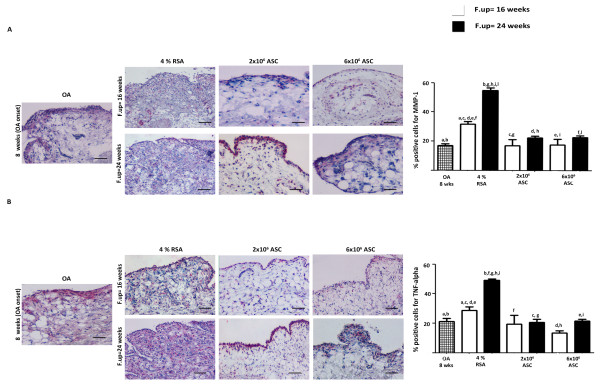
**Immunohistochemical analysis for MMP-1 and TNF-α in synovial membrane**. Photomicrographs of representative specimens evaluated for MMP-1 (**A**) and TNF-α (**B**) in synovial membrane of OA, 4% RSA and ASC-treated groups. Data are reported as mean ± SD. Scale bars = 100 μm. Statistical values of at least *P *< 0.01 were observed in all the comparisons.

**Figure 11:**
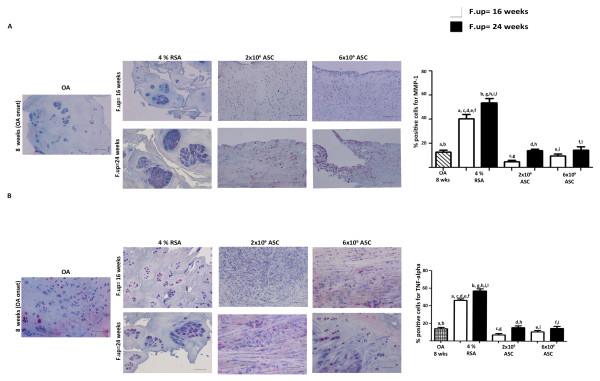
**Immunohistochemical analysis for MMP-1 and TNF-α in menisci**. Photomicrographs of representative specimens evaluated for MMP-1 (**A**) and TNF-α (**B**) in medial menisci of OA, 4% RSA and ASC-treated groups. Data are reported as mean ± SD. Scale bars = 100 μm. Statistical values of at least *P *< 0.01 were observed in all the comparisons.

The effect of ASC treatment on synovial membrane and menisci was investigated by immunohistochemical analyses. The OA group displayed a mild positivity for MMP-1, particularly in the lining layer of the synovial membrane at eight weeks. A moderate expression of MMP-1 was noticed in the 4% RSA group at short- and long-term follow-ups compared to the OA group (*P *< 0.01). ASC treatment reduced the expression for MMP-1 at both experimental times with respect to the 4% RSA group (*P *< 0.01) (Figure 10A). As concerns TNF-α, the OA group showed a mild positivity in the synovial lining at eight weeks. An intense positivity for TNF-α was observed in the 4% RSA at 16 and 24 weeks with respect to the OA group (*P *< 0.01). ASC treatment decreased the expression of TNF-α compared to the 4% RSA group at 16 and 24 weeks (*P *< 0.01) (Figure 10B). A series of investigations was also focused on the status of medial meniscus. The OA group showed a moderate positivity for MMP-1 at the cellular level, particularly in the superficial layer in the meniscal compartment at eight weeks. An increase of MMP-1 expression was noticed in the 4% RSA group at 24 weeks compared to the OA group (*P *< 0.01). By contrast, a reduction of MMP-1 was observed in the ASC-treated groups at 16 weeks compared to the OA group (*P *< 0.01). A slight increase in the expression of this protein was detected in all the groups analyzed at 24 weeks compared to 16 weeks (*P *< 0.01) (Figure 11A). The OA group showed a moderate positivity for TNF-α at the cellular level at eight weeks. The 4% RSA group displayed a strong expression of TNF-α at 16 and 24 weeks with respect to the OA group (*P *< 0.01). A slight positivity was detectable in the ASC-treated groups at both experimental times compared to the 4% RSA group (*P *< 0.01) (Figure 11B).

## Discussion

The employment of ASCs in regenerative medicine is a rapidly growing area of research and some evidence of therapeutic success using these cells for osteochondral defect has been reported [39,40]. Recently, this cell therapy is being used as a valid therapeutic tool also in the treatment of OA. Beneficial effects of ASCs were reported in the care of this pathology in some experimental animal models [25-28]. However, most of these studies were limited to macroscopic and histological observations of articular cartilage after ASC treatment. No information was provided on the effects of these cells on catabolic and inflammatory processes which occur during OA in synovial membrane and menisci. This study, using a rabbit model of OA, was designed to determine the role of ASCs in the OA setting and their behavior on inflammatory environment within the affected articular joint. The ACLT model proposed is widely validated in investigating OA disease, because it determines biomechanical and pathological changes similar to those seen in humans [29]. It has been reported from our group that eight weeks after ACLT, cartilage damages in rabbits occur mainly in the medial femoral condyle, showing a wide spectrum of OA changes, including fibrillation and delamination processes, altered cellular arrangement and proteoglycan depletion [30]. The investigations performed on synovial membrane and menisci revealed that the ACLT procedure at eight weeks leads to a thickening of the lining layer in the synovial membrane associated with the presence of some inflammatory elements and to an increase in cell cluster formation in menisci. Direct intra-articular injection of cells is technically the simplest approach to the use of cells for OA therapy [13,41]. In the current study, an intra-articular injection of ASCs was delivered in the hind limbs of the rabbits after OA induction. No animals showed swelling at the injection sites, signs of distress, or hyperalgesia after ASC administration. There were significant overall effects of ASC treatment in the cartilaginous, synovial and meniscal tissues at different levels. Our investigations gave evidence of the beneficial effect for the 2 × 10^6 ^ASC group, particularly at 16 weeks, showing a well-organized tissue with a low Laverty's score, an increased cartilage thickness compared to the OA group. Both cell doses provided good results showing a high expression of type II collagen in the cartilage matrix at 16 and 24 weeks. A positive contribution of the intra-articular delivery of both ASC concentrations was also noticed in the meniscal compartment at both experimental times, leading to a decrease of the number of cell clusters the fibro-cartilaginous area. Cell treatment inhibits the progression of OA, providing a reduction of the fibrillation index, Laverty's score and type I collagen in cartilage and favors the anabolic processes addressed to the formation of a new tissue. Moreover, ASC administration inhibits the development of thickening of the lining layer in the synovial membrane with major evidence at short-term follow-up. Since the release of cartilage matrix proteins in the articular environment contribute to cartilage damage through the production of inflammatory cytokines, chemokines and MMPs [3], we decided to test the effects exerted by ASCs. To this regard, clear benefits of ASC treatment were observed with the reduction of the inflammation in cartilage tissue in terms of a decreased pattern of expression for TNF-α at both experimental times [42]. In close correlation with the reduction of TNF-α, a decreased level of MMP-1 in cartilage tissue, responsible of proteoglycan degradation, was noticed in ASC-treated groups at 16 and 24 weeks [42,43]. A time-dependent effect of ASC treatment was noticed for MMPs and TNF-α expression, particularly in the 2 × 10^6 ^ASC group providing the best results at short-term follow-up. Cell treatment inhibits the progression of OA, leading to a reduction of TNF-α and MMPs in menisci and synovial membrane at both experimental times. In general, the 4% RSA group displayed OA progression in the different compartments as indicated by an increased expression of catabolic and inflammatory markers. Both ASC doses gave evidence of the healing potential in cartilage, synovial membrane and menisci, even if the lowest cell concentration is more effective on cartilage repair at 16 weeks. The underlying mechanisms responsible of the best effects of 2 × 10^6 ^ASCs in cartilage are not fully understood. It could be related to the release of cytokines and other growth factors by ASCs that at low and high concentrations can determine contrasting biological effects on the immune system and/or on catabolic and inflammatory events. A time-dependent effect by ASCs was observed in the analysis performed particularly on cartilage tissue, detecting the best findings at short-term follow-up. These findings could be justified by the lack of repair of the anterior cruciate ligament, which could slow and/or inhibit some signaling pathways involved in the repair processes. The investigations performed on the local bio-distribution of ASCs by using a fluorescent tracking dye (CM-Dil) open some prospectives in the understanding of the mechanism of action by ASCs. The key advantage of CM-Dil and its derivatives is to represent a nontoxic fluorescent tracking system ready in a few hours, able to label ASCs without altering their multi-potential nature as observed by *in vitro *differentiation protocols and avoiding genetic manipulation of the cells [33]. Nevertheless, this system shows some disadvantages such as the loss of fluorescence signal over time during cell replication rendering difficult the cell tracking at long term follow-up, the transfer of the fluorescent dye to other cells.

After having injected labeled cells, no engraftment was noticed in the anterior cruciate ligament and in the cartilaginous tissue from the tibial plateau and femoral condyles. The homing of these cells was unexpectedly detected in the synovial and medial meniscus, probably due to the expression of specific receptors or ligands able to facilitate trafficking, adhesion and infiltration of ASCs to these sites and/or to the presence of a vascular fraction which could promote ASC migration. The distribution of ASCs in the lining layer of synovial tissue could also be due to the release of chemokines by macrophages located within the lining layer [44]. Different hypotheses could be provided to explain the chondro-protective and healing effects exerted by ASCs. Cell administration could contribute to enhance anabolic signaling pathways and inhibit catabolic ones. These processes could be reasonably induced by growth factors and cytokines released by ASCs rather than their differentiation potential since biodistribution data showed the localization of ASCs in the synovial and meniscal tissues.

Another mechanism exerted by ASCs could be due to the inhibition of the release of catabolic and inflammatory molecules by macrophages in synovium or chondrocytes in cartilage. Experimental evidence supports this hypothesis, reporting a decrease of the catabolic and inflammatory molecules predominantly in cartilage. Other possibilities could be projected on the regulation of the immune system by ASCs as already observed for MSC [37,45]. Several authors have shown how the inflammatory environment is an important parameter to consider, since it would seem to influence the behavior of ASCs by enhancing their immunosuppressive potential [46-49]. This last consideration provides more attention on the pattern of molecules secreted by ASCs, which could have important implications in the resolution of inflammatory diseases. Further analysis of the molecules secreted by ASCs could be useful to identify key elements involved in repair processes. In conclusion, the findings of this study demonstrate that an intra-articular injection of ASCs exerts a chondro-protective role promoting a series of anabolic processes in order to allow the maintenance of a good collagen and proteoglycan network, and at the same time inhibiting catabolic events responsible for degenerative events in cartilage, synovial and meniscal tissues. ASC therapy could, therefore, represent a novel therapeutic tool for the treatment of osteoarthritis.

## Conclusions

The current study was the first to focus on the effect of ASCs in the OA setting and their behavior on the inflammatory environment within the cartilaginous, synovial and meniscal tissues in a rabbit model of osteoarthritis. Our data demonstrated a healing effect by ASCs on cartilage and menisci and an inhibition of OA progression in synovial membrane. Biodistribution of ASCs in medial meniscus and synovium provides some suggestions on their possible role suggesting a paracrine mechanism of action.

## Abbreviations

ACLT: anterior cruciate ligament transection; ASCs: adipose stem cells; BMP-4: bone morphogenetic protein 4; CI: 95% confidence intervals; CM-Dil: chloro-methyl-benzamido-1,1'-dioctadecyl-3,3,3'3'-tetra-methyl-indo-carbocyanine per-chlorate; CT: cartilage thickness; ECM: extracellular matrix; FI: fibrillation index; GLM: general linear model; MMPs: metalloproteinases; MSC: mesenchymal stem cells; OA: osteoarthritis; PD: population doublings; RSA: rabbit serum albumin; RT: room temperature; SD: standard deviation; SVF: stromal vascular fraction; TGF-β1: transforming growth factor-β1; TNF-α: tumor necrosis factor-α

## Competing interests

The authors declare that they have no competing interests.

## Authors' contributions

BG, AF, MF and RG contributed to research design and analyzed data. GD and BG performed the local biodistribution analysis. GD and CC performed cell cultures, while GD and FS performed histological and immunohistochemical analyses. BG, GD and FS performed scoring systems and image analyses. AP and FV performed histomorphometric analyses. MF and RG performed the ACLT procedure. LM performed the anesthesia procedures and the administration of cells into the rabbit knee joint. All authors have read and approved the final manuscript for publication.
